# Large-scale cell production of stem cells for clinical application using the automated cell processing machine

**DOI:** 10.1186/1472-6750-13-102

**Published:** 2013-11-15

**Authors:** Daisuke Kami, Keizo Watakabe, Mayu Yamazaki-Inoue, Kahori Minami, Tomoya Kitani, Yoko Itakura, Masashi Toyoda, Takashi Sakurai, Akihiro Umezawa, Satoshi Gojo

**Affiliations:** 1 Department of Regenerative Medicine, Kyoto Prefectural University of Medicine, 465 Kajii-cho, Kawaramachi-Hirokoji, Kamigyo-ku, Kyoto 602-8566, Japan; 2 System Technology Development Center, Kawasaki Heavy Industries, Ltd., 3-1-1 Higashi Kawasaki-cho, Chuo-ku, Kobe 650-8670, Japan; 3 Department of Reproductive Biology and Pathology, National Center for Child Health and Development, 2-10-1 Okura, Setagaya-ku, Tokyo 157-8535, Japan; 4 Department of Cardiovascular Medicine, Kyoto Prefectural University of Medicine, 465 Kajii-cho, Kawaramachi-Hirokoji, Kamigyo-ku, Kyoto 602-8566, Japan; 5 Department of Vascular Medicine, Tokyo Metropolitan Institute of Gerontology, 35-2 Sakae-cho, Itabashi-ku, Tokyo 173-0015, Japan

**Keywords:** Automated cell culture system, Cell transplantation, Stem cells, Clinical trial, Cell processing facility

## Abstract

**Background:**

Cell-based regeneration therapies have great potential for application in new areas in clinical medicine, although some obstacles still remain to be overcome for a wide range of clinical applications. One major impediment is the difficulty in large-scale production of cells of interest with reproducibility. Current protocols of cell therapy require a time-consuming and laborious manual process. To solve this problem, we focused on the robotics of an automated and high-throughput cell culture system. Automated robotic cultivation of stem or progenitor cells in clinical trials has not been reported till date. The system AutoCulture® used in this study can automatically replace the culture medium, centrifuge cells, split cells, and take photographs for morphological assessment. We examined the feasibility of this system in a clinical setting.

**Results:**

We observed similar characteristics by both the culture methods in terms of the growth rate, gene expression profile, cell surface profile by fluorescence-activated cell sorting, surface glycan profile, and genomic DNA stability. These results indicate that AutoCulture® is a feasible method for the cultivation of human cells for regenerative medicine.

**Conclusions:**

An automated cell-processing machine will play important roles in cell therapy and have widespread use from application in multicenter trials to provision of off-the-shelf cell products.

## Background

Degenerative diseases affect increasing numbers of people, particularly in developed countries with aging populations. Despite advancements in medicine, modalities to cure advanced diseases are often not available. Therefore, regenerative therapy may become the standard treatment option in cardiovascular medicine. Recent developments in stem cell biology, including those related to induced pluripotent stem cells (iPSCs) and tissue-derived stem/progenitor cells, are a giant leap toward the goal. Recently, myocardium-derived stem/progenitor cells were isolated by several institutes [1-3]. These cell populations have the potential to repair the diseased heart, and clinical trials are currently ongoing.

In tandem with these developments in stem cell biology and the large number of completed and ongoing clinical trials, attempts have been made to commercialize these therapies [4]. The most prominent therapeutic strategy is cell transplantation. However, harvested cells or tissues are usually limited in quantity and stem cells properties may vary from batch to batch, hindering the reliability for clinical applications. Moreover, current cell therapy protocols are laboratory centered and labor intensive, requiring highly skilled personnel and weeks to months to harvest sufficient quantities of stem/progenitor cells from the isolated tissues. These manual procedures are expensive and can result in high phenotypic and yield variability between different trials and institutions [5].

**Figure 1:**
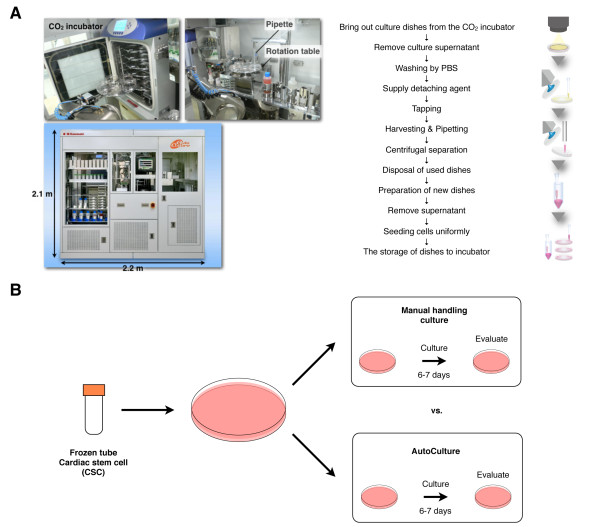
**Process maps showing the key steps in human cardiac stem cell culture using manual methods and the AutoCulture® system. (A)** The automated culture system AutoCulture® by Kawasaki Heavy Industries (left) can automate every step of manual cell culture under current good manufacturing practice (cGMP) grade. **(B)** Schematic representation of the experiment. Human cardiac stem cells (CSCs) were thawed and split into 2 dishes for either manual culture or automated culture using AutoCulture®.

Strategies to validate advanced medicinal products have been established; however, these “best practices” still depend on the ability of personnel to perform them, such as the cultivation of stem/progenitor cells under strictly controlled conditions [6]. High process reproducibility can be achieved by automation, and several effective automatic cell culture systems have been reported [7-12]. These automated platforms have the potential to provide cost-effective, large-scale expansion of stem/progenitor cells with consistent phenotype for clinical use and improved operational safety [13]. Progress in robot platforms for cell culture has resulted in several prototypes to implement large-scale expansion and harvesting of stem/progenitor cells with yield and phenotypic reproducibility. An automated culture system by “The Automation Partnership Biosystems (TAP Biosystems)” has cultivated human embryonic stem cells and bone marrow-derived cells [14,15]. Kawasaki Heavy Industries (Tokyo, Japan) has created AutoCulture® (Additional file 1), which can automate many manual steps in cell culture, including media exchange, centrifugation of cells, splitting and passaging, and recording of cell morphology (Figure 1A). To the best of our knowledge, no cell products obtained from an automated culture apparatus have actually been transplanted into humans for regenerative therapy.

Our institute recently completed a phase I clinical trial using autologous cardiac stem cells (CSCs) isolated by manual cell culture techniques to treat ischemic cardiomyopathy [16]. The trial is registered in the Japanese government database for clinical trials using human stem cells and ClinicalTrials.gov, which is a world-wide registry and results database for clinical trials involving humans, as AutoLogous Human CArdiac-Derived Stem Cell to Treat Ischemic cArdiomyopathy (ALCADIA; Identifier: NCT00981006). CSCs are manually cultivated by a single experienced investigator for approximately 1 month to minimize variability of the final cell products. To advance this trial from a single-center to a multi-center randomized trial, we evaluated AutoCulture® by comparing the growth rate, morphology, and phenotype of cells cultivated using this method with those of manually cultured CSCs.

## Results

### Cellular morphology and growth

**Figure 2:**
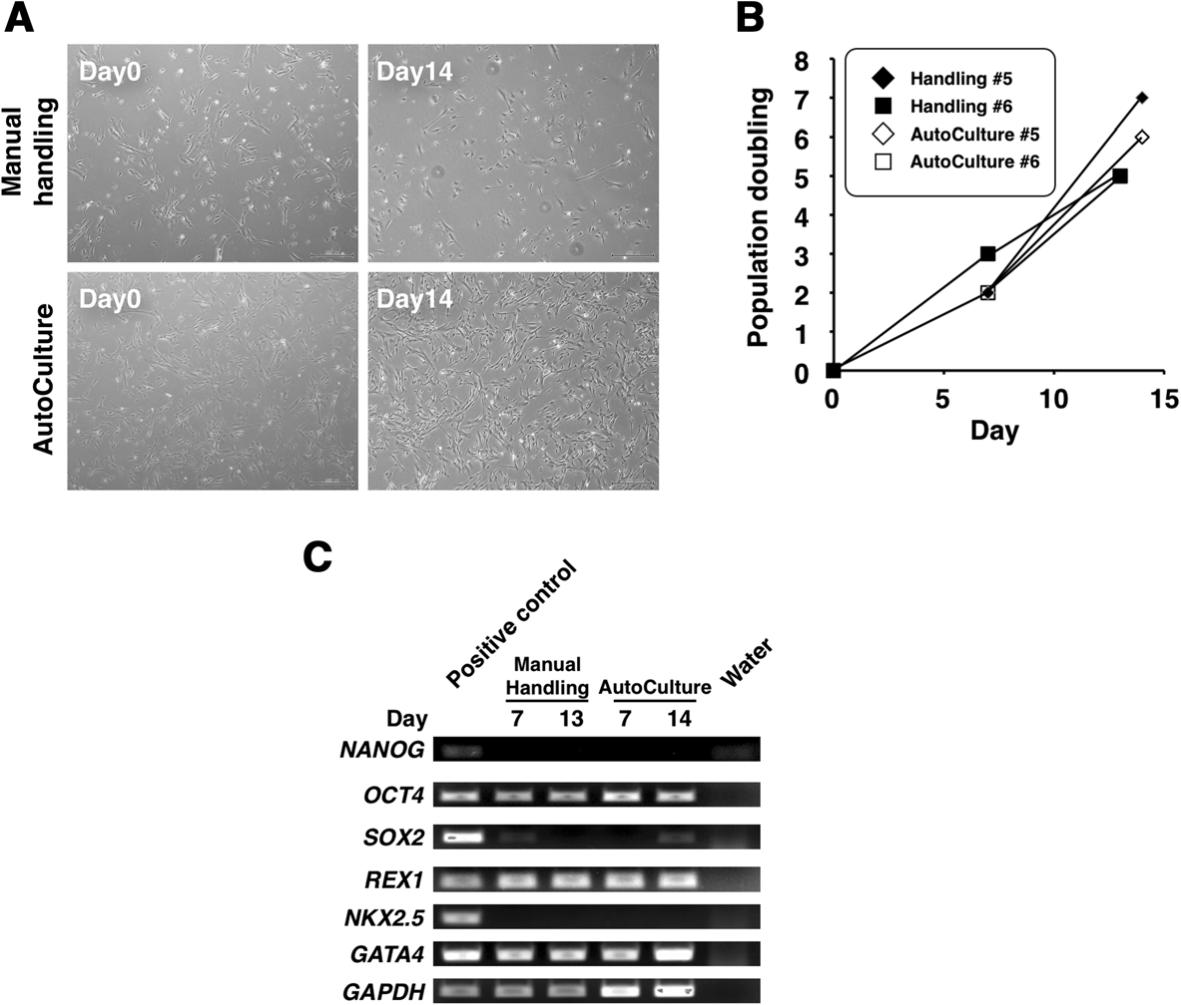
**Phenotypic characterizations of CSCs during manual and automated culture. (A)** Phase contrast microphotographs of CSCs cultivated either by manual handling (upper panels) or using AutoCulture® (lower panels). **(B)** Growth rates of CSCs under manual or automated culture. **(C)** RT-PCR analysis of genes related to pluripotency and cardiac-specific transcription factors.

Calculations based on the net cell number and doubling time obtained in the ALCADIA trial (Additional file 2) indicated that a culture duration of 2 weeks was sufficient to obtain the appropriate cell number for clinical trial when cells after the second passage (P2) were used as the starting material. Identically seeded culture plates were maintained manually or by automation using AutoCulture® (Figure 1B). The morphology of CSCs cultured using the automated system was similar to that of manually cultured CSCs on day 7 and 14 after seeding (Figure 2A). Under both the conditions, the cells were of similar size, exhibited a low nucleus/cytoplasm ratio, and had a spindle-like shape. In addition, the growth rate was not significantly different, as indicated by cell counts at passage (Figure 2B). Trypan blue staining revealed no significant difference in cell viability between the culture methods. Moreover, both the methods effectively washed out the cells, as indicated by the paucity of adherent cells on discarded culture dishes (data not shown). These results suggest that manual passage was effectively replicated using AutoCulture®.

### Gene expression

To investigate the gene expression profiles, RT-PCR analysis was performed according to the shipping criteria for cultivated cells in the current clinical trial (ALCADIA). We examined expression levels of the pluripotency related genes *NANOG*, *OCT4*, *SOX2*, and *REX1* and 2 transcription factor genes involved in cardiomyocyte development, *NKX2*.5 and *GATA4* (Figure 2C). The stem cell markers *OCT4*, *REX1*, and *GATA4* were expressed by both cell populations; however, neither *NANOG* nor *NKX2.5* expression was detectable. Moreover, expression levels were not significantly different between the 2 groups on either day 7 or day 14.

### Cell surface marker expression profiles

**Figure 3:**
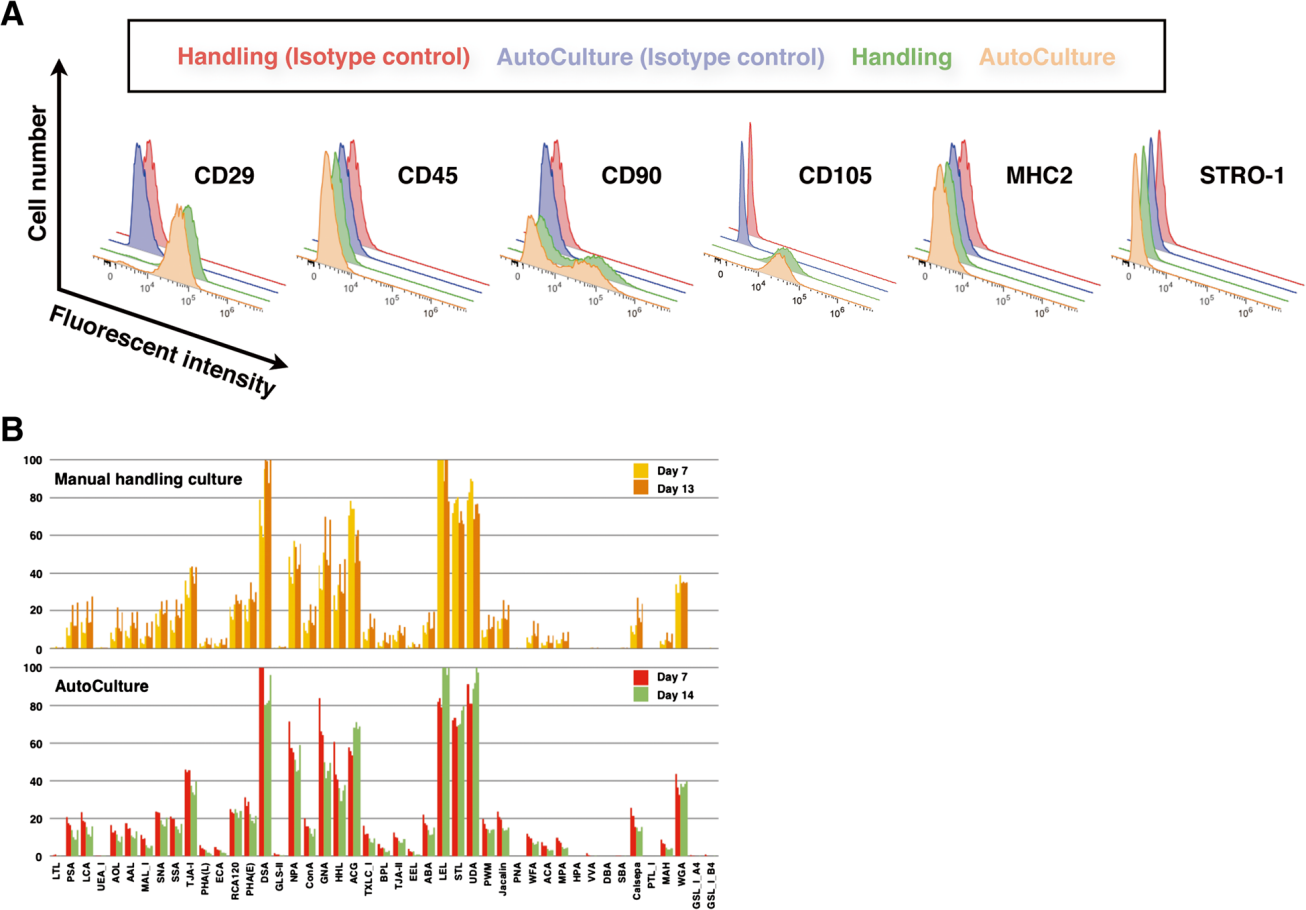
**Analysis of cell surface protein and glycan expression in CSCs expanded by manual culture or AutoCulture®. (A)** Cells were stained with fluorescence-conjugated primary antibodies and analyzed for surface expression by fluorescence-activated cell sorting. **(B)** Lectin microarray analysis of manual cultures and cells maintained using AutoCulture® on day 7 and day 13/14. The glycan profiles showed little difference between the two culture groups at any time.

Cell surface markers indicative of CSCs and other phenotypes were detected by fluorescence-activated cell sorting (FACS) (Figure 3A). Under both the culture conditions, the cells were positive for the mesenchymal stem cell (MSC) markers CD29 and CD90 and the vascular endothelial marker CD105 and negative for the hematopoietic lineage marker CD45 and MHC class II. In addition, fluorescent intensities measured by FACS were similar for all positive markers, indicating that equal proportions of cells in both the populations expressed these proteins. Moreover, almost all the cells were CD29 positive, whereas at least 2 populations were distinguished on the basis of CD90 expression. Furthermore, STRO-1, which is expressed by mesenchymal stem cells in the bone marrow, was negative in both the populations. Although the surface expression profiles of CSCs and bone marrow-derived stem cells overlap, STRO-1 expression can discriminate cardiac MSCs from bone marrow-derived MSCs.

### Surface glycan expression profile by lectin microarray analysis

Recently, glycan expression profiling has been reported to be an effective cell validation tool to complement phenotype analysis by epigenetic and gene expression analyses [17]. These lectin profiles showed similar patterns, and no significant differences in expression intensities were observed between the 2 culture groups on either day 7 or day 13/14 after seeding (Figure 3B). The washing process used to harvest adherent cells may have profound effects on the cell surface structure and expression. CSCs harvested from the AutoCulture® system exhibited similar surface expression profiles and overall viability to those cultured manually.

### Analysis of array comparative genomic hybridization (aCGH) and microarray

**Figure 4:**
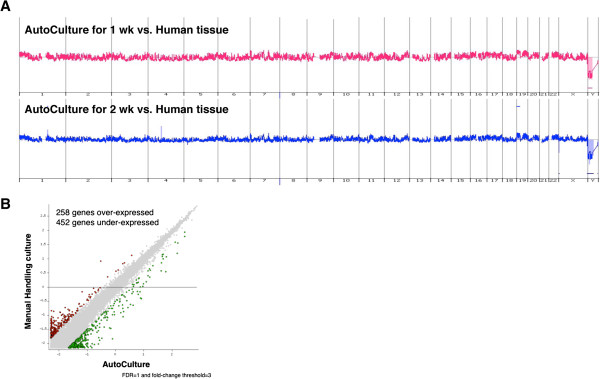
**Global analysis of gene expression and genomic DNA mutations. (A)** Comparison of array comparative genomic hybridization analysis. Human gDNA (from RAA) was compared to gDNA extracted from CSCs cultivated using AutoCulture® after 1 and 2 weeks using CGH arrays (arrays G4413A). **(B)** Microarray analysis of gene expression profiles. The pairwise scatter plot presents differences in gene expression on day 7 after seeding between manually cultured CSCs and those cultured using AutoCulture®. Grey dots represent transcripts with a subthreshold difference in expression. Red or green dots indicate those with at least a 3-fold difference in expression levels between the two groups. Gene expression levels are depicted on a log2 scale. The number of differentially expressed genes is indicated above.

To detect genomic DNA mutations on AutoCulture®, we performed aCGH analysis (Agilent technologies) on day 7 and day 14 and compared them with the tissue derived from human right atrial appendage (RAA) (Figure 4A). There were no differences in genomic DNA mutation between CSCs in AutoCulture® and RAA. To investigate the global gene expression profile changes between CSCs in manual culture and CSCs in AutoCulture®, we performed a pairwise comparison of gene expression microarray data using NIA array analysis [18]. The results revealed a similar gene pattern between them (Figure 4B, Additional file 3). The “Symbol” of 162 gene probes was left blank in 258 overexpressed gene probes.

## Discussion

Cell-based regenerative medicine is still in the early stages of development [19,20]. The quality of cells for transplant depends on the ability of skilled personnel to isolate, expand, and harvest cultured cells. For consistency of cell yield and phenotype, it is imperative that methodological consistency is strictly maintained. Automation greatly enhances the consistency of culture conditions and may thus reduce the variability in cell quality that is one of the great impediments to the widespread application of cell-based therapy till date. In this study, we used the automated cell culture system AutoCulture® to expand human CSCs isolated from the RAA for use in the ALCADIA clinical trial designed to assess the safety of cell-based therapy for patients with ischemic cardiomyopathy. RAA-derived cells containing CSCs exhibited similar growth rates and gene expression profiles between manual and automated cultures. Thus, the AutoCulture® system effectively replicated manual culture and demonstrated scalability and stability in addition to safety and cost-effectiveness. Indeed, we found no significant differences in phenotype between the two culture methods. Cells in both the populations had similar morphologies, mean growth rates, and expression levels of genes associated with pluripotency and the mesenchymal lineage. In addition, the surface glycan profile was virtually identical, while aCGH analysis revealed no difference in genomic DNA mutation frequency. Finally, the approximately 41,000-probe Agilent Whole Human Genome Microarray chip G4112F showed that only approximately 1% of transcripts measured were significantly under- or overexpressed. The successful transfer of manual to automated cell culture may be attributable to the high flexibility of the machine, which can faithfully copy every step and condition, including media changes, splitting, and passaging in a controlled environment.

AutoCulture® is an all-in-one automated cell culture system consisting of robot arms, tube and flask decappers, flask holders, flask tappers, media pumps, a pipette head, a centrifugal separator, a rotating plate, and a CO_2_ incubator. In addition to media change and passage, it permits routine observation. To automate these culture steps, it is necessary to program the humidity, temperature, volume and flow of liquid, and robot arm motion that transfers flasks from or into the CO_2_ incubator or flask holder. Another automated cell culture platform, TAP CompacT, was also shown to be an effective system for culture of adherent cells by the Healthcare Engineering group [14]. However, the lack of a centrifugal separator in that system may result in differences between the manual and automated processes, possibly explaining why automation resulted in a smaller population of STRO-1+ cells and overall lower cell yield after the first passage [21]. STRO-1 expression is not a necessary or specific marker for stem or progenitor cells, and somatic stem cells may be more resistant to nutritional and chemical stress [22]. Residual trypsin in the culture media may have adversely affected the survival of differentiated cells, but it is not clear whether stem or progenitor cells can survive or not. On the other hand, the AutoCulture® system efficiently removes trypsin/EDTA by washing and centrifugation. There were no significant differences in the surface marker expression profile or the mean rate of proliferation between these cells and those maintained manually, strongly suggesting that both populations of RAA-derived CSCs contain equal properties.

The AutoCulture® system can save labor and costs by expanding the scale of production and maintaining uniformity of results. In addition, this system can simultaneously cultivate different cells without cross-contamination because it can be equipped with a connecting hatch to multiple CO_2_ incubators. Large-scale production and multisample cell culture capacity for cell transplantation may be a prerequisite for commercialization of cell products under current good manufacturing practice (cGMP) grade. Production methods for cell therapy should be designed to ensure that the end product is standardized and safe. cGMP is a quality assurance system that ensures that the cell product meets preset specifications with minimal lot-to-lot variability [23]. It requires traceability of raw materials used in cell culture and validated standard operating procedures (SOPs) throughout the process [24,25]. Current good tissue practice (cGTP) is intended to prevent human cells, tissues, and cellular and tissue-based products from contamination by infectious disease agents and to ensure that these cells and tissues maintain their integrity and function. The controlled environment of a carefully designed, constructed, validated, and maintained clean room will minimize the risks of environmental contamination and decrease the possibility of cross-contamination [26]. Based on cGMP, aseptic handling and filling of raw materials should be performed in a grade A environment (class 100) with a grade B background (class 1,000). Clean room disciplines, gowning procedures, cleaning programs, and maintenance of air handling units are included in SOPs. Environmental monitoring is essential in clean room quality control. Proper cleaning, maintenance, repair, and attire are major issues for cGMP [27].

Construction and maintenance of a cGMP facility is so expensive that it may be difficult to conform to these standards on a large scale without automation. Unlike manual culture, the robots enabled the environment in the cell culture cabinet to be completely separated from the external environment. Moreover, automated cell culture machines can be equipped with cleaning and monitoring systems to prevent contamination by microorganisms and cross-contamination by other cell types cultured in tandem. These properties may meet the stringent conditions for a human cell processing facility while reducing both construction and maintenance costs.

In Japan, the regulatory path of a regenerative cell therapy using this automated machine will be to obtain an approval for the end products, such as cells or tissues, based on the new guidelines and philosophy at an initial phase. An important requirement for obtaining approval is publication of the safety and reliability of the machine to produce the final biological products in a peer-reviewed journal. The similar properties of cell products between those obtained by machine and those obtained by manual culture, as demonstrated in this study, could support approval of a clinical trial using this machine, which is currently being planned.

## Conclusion

AutoCulture® is one of the best candidates to solve the problems inherent in large-scale production and harvesting of human cells for clinical applications. The automated cell processing system can reproduce many complex operations performed by professional staff and can maintain multiple cell lines automatically. Thus, this automation system will be a powerful tool for both clinical trials exploring the potential of autologous or allogeneic cell-based regeneration therapies and for the commercialization.

## Methods

### Isolation of human CSCs containing atrial appendage

After this study was approved by the ethics committees of Tokyo Metropolitan Geriatric Hospital (ID: #220106), human cardiac tissue samples from RAA were surgically excised from 7 patients (60–75 years old) during cardiac surgery. All patients provided written informed consent. A cell population containing CSCs was acquired according to the current protocol for ALCADIA [28]. In brief, the tissue fragments were cut into 5 × 5-mm pieces and incubated with 0.2% collagenase type II and 0.1% DNase I (Worthington Biochemicals) at 37°C for 30 min. The cells were cultivated in a basic culture medium of Dulbecco’s modified Eagle medium (DMEM)/F12 supplemented with 10% fetal bovine serum (FBS) and 40 ng/ml basic fibroblast growth factor (bFGF). The cells were seeded in 60-mm dishes coated with collagen type I. The cultured cells were passaged twice, harvested, and frozen until used in this experiment. P2 cell population was utilized as the starting material for this comparison experiment.

### Cell expansion and harvesting

After thawing, the cells derived from the human atrium were seeded at 1 × 10^5^ cells per 100-mm culture dish and cultivated for 5–7 days. The cells were split at 1:10 at 80%–90% confluence. The basic culture medium was replaced every 3 or 4 days. For automated culture, we used the same lot of CSCs. After seeding, the culture dishes were placed in the AutoCulture® chamber and transferred into the internal CO_2_ incubator by the robot arm (Figure 1A, Additional file 4). For media replacement, the robot arm retrieved the culture dishes from the incubator and set them on a rotating table. The dish covers were removed by the robot arm, a specified amount of medium was discarded, fresh medium was supplied by a new pipette, the covers were returned, and the culture dishes was transferred back to the CO_2_ incubator. For passage, the old medium was removed and DPBS was pipetted onto the dishes under gentle shaking. After washing in DPBS, AutoCulture® supplied trypsin, oscillated the culture dishes, and returned them to the CO_2_ incubator for a 5-min incubation. Following this, the robot arm moved the culture dishes onto the rotation table, added a prespecified volume of the basic culture media, and transferred the cell suspension from each dish to a separate 50-ml centrifuge tube. The cell suspension was centrifuged at 200 × *g* for 5 min at room temperature, and the supernatant was discarded. Fresh basic culture medium was supplied to the cell pellet, which was then resuspended. The washed cell suspension was subcultured at approximately 1:10 onto new culture dishes and returned to the CO_2_ incubator.

### Reverse transcription-polymerase chain reaction (RT-PCR)

Total RNA was extracted from cell populations containing CSCs, from human iPSCs, and raw human heart tissue samples using the RNeasy Plus Mini Kit (QIAGEN) as positive/negative control. Total RNA from human iPSCs and the human heart (Clontech Laboratories) was used as the positive control for each primer. Total RNA (500 ng per reaction) was converted to cDNA using the Transcriptor High Fidelity cDNA Synthesis Kit (Roche Applied Science) according to the manufacturer’s protocol. Primers for the cardiac-specific transcription factors *NKX2.5* and *GATA4*; the stem cell markers *NANOG*, *OCT3/4*, *SOX2*, and *REX1*; and the housekeeping gene *GAPDH* were obtained from PrimerBank (Additional file 5).

### Flow cytometric analysis

The cells (1 × 10^6^ per reaction) were stained in autoMACS Running Buffer (Miltenyi Biotec.) with fluorescence-conjugated primary antibodies for 30 min at 4°C. The cells were then analyzed on the Attune Acoustic Focusing Cytometer (Applied Biosystem), and the data were analyzed using FlowJo 8.8.7 software (TOMY Digital Biology). Antibodies used for phenotyping included anti-human CD29-PE, CD90-PE, CD105-FITC, STRO-1-FITC, CD45-PE, and MHC class II-PE. Isotype controls were FITC-conjugated mouse IgG_1_, PE-conjugated mouse IgG_1_, and FITC-conjugated mouse IgM.

### Lectin microarray analysis

Proteins were extracted from each cell population in hydrophobic and hydrophilic fractions using the CelLytic MEM Protein Extraction Kit (Sigma-Aldrich), as described previously [29]. Lectin microarray analysis was performed as described previously, with only minor modifications [30]. The glycoprotein (200 ng) was labeled with Cy3 mono-reactive dye (GE Healthcare) in DPBS containing 0.5% Triton X-100 (PBSTx) at room temperature for 1 h. The Cy3-labeled glycoprotein solution (60 μl) was applied to the LecChip (GP Bioscience), which has triplicate spots specific for 45 lectins on each glass slide. An evanescent-field fluorescence scanner (GlycoStationTM Reader) was used to analyze the LecChip. All data were analyzed with GlycoStationTM Tools Signal Capture 1.0 and GlycoStationTM Tools Pro 1.0 software (GP Bioscience). To expand the dynamic range, the data were subjected to a gain-merging procedure, and the merged data were then normalized with max-normalization, as described previously [29].

### aCGH analysis

Genomic DNA from the heart tissue and cultured cells was isolated using the DNeasy Blood & Tissue Kit (QIAGEN). Labeled test and reference DNAs were combined, denatured, preannealed with Cot-1 DNA (Invitrogen) and blocking agent, and then hybridized to the arrays (SurePrint G3 Human CGH Microarray 2x400K, Agilent Technologies). After hybridization and washing, the arrays were scanned at 3-μm resolution using an Agilent G2505C scanner. Images were analyzed with Feature Extraction software 10.7.3.1 (Agilent Technologies) using the CGH 107 Sep09 protocol for background subtraction and normalization.

### Gene expression analysis

Gene expression analysis was performed using the Agilent Whole Human Genome Microarray chip G4112F (Agilent Technologies), which contains >41,000 probes. Raw data were normalized and analyzed by GeneSpring GX11 software (Silicon Genetics). Pairwise scatter plot analysis of the global gene expression profiles of both manually cultured cells and autocultured cells was performed on day 7 after thawing. The number of differentially expressed genes is indicated over each scatter plot. The NIA Array [18] web tool was used for pairwise scatter plot analysis. Gene expression microarray data have been submitted under accession number GSE 44032. Analysis of microarray experiments was conducted using the Aberration Detection Method-2 statistical algorithm (Agilent Technologies) on the basis of the combined log2 ratios at a threshold of 6.0. The data were centralized, and calls with average log2 ratios <0.3219 were filtered to exclude false positives.

## Abbreviations

CSC: Cardiac stem cells; FACS: Fluorescence-activated cell sorting; MSCs: Mesenchymal stem cells; aCGH: Array comparative genomic hybridization; RAA: Right atrial appendage; cGMP: Current good manufacturing practice; SOPs: Standard operating procedures; cGTP: Current good tissue practice; bFGF: Basic fibroblast growth factor.

## Competing interests

DK, MYI, KM, TK, YI, MT, AU and SG declare that they have no competing interests. KW and TS are employees of Kawasaki Heavy Industries, Ltd.

## Authors’ contributions

DK, MT, AU, and SG designed the research; DK, KW, YI, KM, MYI, and performed the experiments; DK, MT, and SG analyzed the data; and DK, TK, YI, and SG wrote the manuscript. All authors read and approved the final manuscript.

## Supplementary Material

Additional file 1 **Document 1.** Specialization of the automated cell processing machine (Auto Culture®). Click here for file

Additional file 2 **Document 2.** Quantitative cellular aspects for ALCADIA clinical trial. Click here for file

Additional file 3: Table S1 Results of microarray analysis of CSCs in manual culture and AutoCulture®. To investigate the differences in global gene expression profile between CSCs in manual culture and CSCs in AutoCulture®, we performed a pairwise comparison of gene expression microarray data using NIA array analysis. The results revealed similar gene expression patterns between them. Click here for file

Additional file 4 **Movie 1.** AutoCulture®.Movie of the culture robot in AutoCulture®. Click here for file

Additional file 5: Table S2 RT-PCR primer sequences. RT-PCR primer sequences were obtained from PrimerBank (http://pga.mgh.harvard.edu/primerbank/). Click here for file

## References

[B1] Takehara N Tsutsumi Y Tateishi K Ogata T Tanaka H Ueyama T Takahashi T Takamatsu T Fukushima M Komeda M Controlled delivery of basic fibroblast growth factor promotes human cardiosphere-derived cell engraftment to enhance cardiac repair for chronic myocardial infarction J Am Coll Cardiol 2008 13 23 1858 1865 10.1016/j.jacc.2008.06.052 19038683

[B2] Smith RR Barile L Cho HC Leppo MK Hare JM Messina E Giacomello A Abraham MR Marban E Regenerative potential of cardiosphere-derived cells expanded from percutaneous endomyocardial biopsy specimens Circulation 2007 13 7 896 908 10.1161/CIRCULATIONAHA.106.655209 17283259

[B3] Rota M Padin-Iruegas ME Misao Y De Angelis A Maestroni S Ferreira-Martins J Fiumana E Rastaldo R Arcarese ML Mitchell TS Local activation or implantation of cardiac progenitor cells rescues scarred infarcted myocardium improving cardiac function Circ Res 2008 13 1 107 116 10.1161/CIRCRESAHA.108.178525 18556576 PMC2747796

[B4] Vogel G Stem cells for sale Science 2010 13 6008 1173 10.1126/science.330.6008.1173 21109646

[B5] Tran CA Burton L Russom D Wagner JR Jensen MC Forman SJ DiGiusto DL Manufacturing of large numbers of patient-specific T cells for adoptive immunotherapy: an approach to improving product safety, composition, and production capacity J Immunother 2007 13 6 644 654 10.1097/CJI.0b013e318052e1f4 17667528

[B6] Soncin S Lo Cicero V Astori G Soldati G Gola M Surder D Moccetti T A practical approach for the validation of sterility, endotoxin and potency testing of bone marrow mononucleated cells used in cardiac regeneration in compliance with good manufacturing practice J Transl Med 2009 13 78 10.1186/1479-5876-7-78 19737416 PMC2753319

[B7] Joannides A Fiore-Heriche C Westmore K Caldwell M Compston A Allen N Chandran S Automated mechanical passaging: a novel and efficient method for human embryonic stem cell expansion Stem Cells 2006 13 2 230 235 10.1634/stemcells.2005-0243 16510428

[B8] Kino-Oka M Ogawa N Umegaki R Taya M Bioreactor design for successive culture of anchorage-dependent cells operated in an automated manner Tissue Eng 2005 13 3–4 535 545 15869432 10.1089/ten.2005.11.535

[B9] Terstegge S Laufenberg I Pochert J Schenk S Itskovitz-Eldor J Endl E Brustle O Automated maintenance of embryonic stem cell cultures Biotechnol Bioeng 2007 13 1 195 201 10.1002/bit.21061 16960892

[B10] Thomas RJ Anderson D Chandra A Smith NM Young LE Williams D Denning C Automated, scalable culture of human embryonic stem cells in feeder-free conditions Biotechnol Bioeng 2009 13 6 1636 1644 10.1002/bit.22187 19062183

[B11] Koike H Kubota K Sekine K Takebe T Ouchi R Zheng YW Ueno Y Tanigawa N Taniguchi H Establishment of automated culture system for murine induced pluripotent stem cells BMC Biotechnol 2012 13 81 10.1186/1472-6750-12-81 23127273 PMC3499150

[B12] Thomas RJ Chandra A Liu Y Hourd PC Conway PP Williams DJ Manufacture of a human mesenchymal stem cell population using an automated cell culture platform Cytotechnology 2007 13 1 31 39 10.1007/s10616-007-9091-2 19002992 PMC2289788

[B13] Hubbell JA Palsson BO Papoutsakis ET Preface: tissue engineering and cell therapies: II Biotechnol Bioeng 1994 13 8 683 10.1002/bit.260430802 18615791

[B14] Thomas R Chandra A Hourd P Williams D Cell culture automation and quality engineering: a necessary partnership to develop optimized manufacturing processes for cell-based therapies J Assoc Lab Autom 2008 13 3 152 158 10.1016/j.jala.2007.12.003

[B15] Thomas RJ Hourd PC Williams DJ Application of process quality engineering techniques to improve the understanding of the in vitro processing of stem cells for therapeutic use J Biotechnol 2008 13 3–4 148 155 18672011 10.1016/j.jbiotec.2008.06.009

[B16] Takehara N Ogata T Nakata M Kami D Nakamura T Matoba S Gojo S Sawada T Yaku H Matsubara H The ALCADIA (Autologous Human Cardiac-Derived Stem Cell to Treat Ischemic Cardiomyopathy) trial Circulation 2012 PHILADELPHIA, PA: LIPPINCOTT WILLIAMS & WILKINS 530 WALNUT ST 19106 3621 USA: 2783–2783

[B17] Toyoda M Yamazaki-Inoue M Itakura Y Kuno A Ogawa T Yamada M Akutsu H Takahashi Y Kanzaki S Narimatsu H Lectin microarray analysis of pluripotent and multipotent stem cells Genes Cells 2011 13 1 1 11 10.1111/j.1365-2443.2010.01459.x 21155951

[B18] Sharov AA Dudekula DB Ko MS A web-based tool for principal component and significance analysis of microarray data Bioinformatics 2005 13 10 2548 2549 10.1093/bioinformatics/bti343 15734774

[B19] Ptaszek LM Mansour M Ruskin JN Chien KR Towards regenerative therapy for cardiac disease Lancet 2012 13 9819 933 942 10.1016/S0140-6736(12)60075-0 22405796

[B20] Gojo S Toyoda M Umezawa A Tissue engineering and cell-based therapy toward integrated strategy with artificial organs J Artif Organs 2011 13 3 171 177 10.1007/s10047-011-0578-4 21660420

[B21] Liu Y Hourd P Chandra A Williams DJ Human cell culture process capability: a comparison of manual and automated production J Tissue Eng Regen Med 2010 13 1 45 54 19842115 10.1002/term.217

[B22] Kuroda Y Kitada M Wakao S Nishikawa K Tanimura Y Makinoshima H Goda M Akashi H Inutsuka A Niwa A Unique multipotent cells in adult human mesenchymal cell populations Proc Natl Acad Sci USA 2010 13 19 8639 8643 10.1073/pnas.0911647107 20421459 PMC2889306

[B23] Unger C Skottman H Blomberg P Dilber MS Hovatta O Good manufacturing practice and clinical-grade human embryonic stem cell lines Human Mol Genet 2008 13 R1 R48 R53 10.1093/hmg/ddn079 18632697

[B24] Allport-Settle MJ Good Manufacturing Practice (GMP) Guidelines 2009 Raleigh: Pharmalogika

[B25] Burger SR Current regulatory issues in cell and tissue therapy Cytotherapy 2003 13 4 289 298 10.1080/14653240310002324 12944234

[B26] USP 32-NF 27 Cell and gene therapy products 2008 United States Pharmacopeia, Rockville: Manufacturing of cell therapy products

[B27] Niazi SK Sterile products, vol. 6 2009 London: Informa Healthcare Inc

[B28] Takehara NOT Nakata M Kami D Matoba NT Gojo S Sawada T Yaku H Matsubara H The ALCADIA (autologous Human Cardiac-derived Stem Cell To Treat Ischemic Cardiomyopathy) Trial Late Breaking Clinical Trial Application 2012 Los Angeles: American Heart Association, Scientific Session

[B29] Kuno A Itakura Y Toyoda M Takahashi Y Yamada M Umezawa A Hirabayashi J Development of a data-mining system for differential profiling of cell glycoproteins based on lectin microarray J Proteom Bioinfo 2008 13 2 5

[B30] Itakura Y Kimura M Gojo S Toyoda M Kami D Motomura N Umezawa A Kyo S Ono M Glycan profiling using a lectin microarray is a novel validation tool for monitoring the damage to freeze-thawed cells Low Temp Med 2011 13 7

