# Supplementary material for: Large-scale cell production of stem cells for clinical application using the automated cell processing machine
Source: BMC Biotechnol. 2013 Nov 15;13:102. doi: 10.1186/1472-6750-13-102 (PMC4101824; doi:10.1186/1472-6750-13-102)

Additional Document 1

**Specialization of the Automated Cell Processing Machine (Auto Culture^®^)**

The automated cell processing machine is controlled by 5 types of softwares that communicate with each other to perform all operations with high reliability (Additional figure 1).

To prevent contamination, including microbial and sample cross-contamination, the following precautions are taken: (i) Disposable tips are used for supply and removal of reagents (Additional figure 2A). (ii) A clean class 100 environment is maintained within the machine cabinet by downward air flow through a HEPA filter system, which provides protection from air turbulence due to machine operation, given that the cleanliness level of the environment outside the machine is approximately clean class 100,000. (iii) The HEPA filter system normally operates in a low-flow mode. The operation mode is automatically changed to a forced ventilation mode when the cleanliness class within the machine cabinet is increased, when robotic movements are in operation, or when the doors of the machine cabinet are opened for shipping. (iv) The clean class inside the machine cabinet is constantly monitored and the data is recorded. In addition, the system sends out a warning when the clean class exceeds 100. (v) The machine cabinet is maintained at a positive pressure, preventing invasion of any particle from the outside. (vi) An alcohol spray is used to sterilize the cabinet after each set of culture operations per culture batch. (vii) A UV lamp is turned on regularly after operation. (viii) Simultaneous culture of fluorescently labeled and non-labeled cells for a period of time has confirmed no occurrence of cross-contamination.

Mistakes in sample collection are prevented by labeling, which gives necessary and sufficient information to identify each sample. The information consists of the name of the cells, the passage number, the cell number, the volume, the date of shipping, and the lot number (Additional figure 2B).

A warning system is set to send out emails notifying occurrence of abnormalities in several types of monitoring parameters (as described in the following section), such as temperature and humidity (Additional figure 2C). A remote maintenance function allows swift response and handling by the system manager. In case of a power outage, control PCs, robotics, and CO_2_ incubators are backed up with uninterruptable power supplies. A warning system can be set to send out emails notifying occurrence of a power outage.

The following data are recorded continuously by the system: clean class, pressure difference, temperature, and humidity in the main machine cabinet; temperature, CO_2_ concentration, and amount of humidification water in the incubator; temperature in the refrigerated storage; and device operation status. Additional figure 2D shows a trend chart of temperature in the machine cabinet.

The cellular morphology and density can be observed using a phase contrast microscope, which is located in the main cabinet, by way of an external monitor display (Additional figure 3A and B). The display is also used as a surveillance monitor and for all operating softwares.

Cell passage operation is performed by one of the following 3 ways: a preregistered schedule, user’s decision, or automatic determination. In case of user’s decision, an operator determines the best timing for passage on the basis of the information displayed on the monitor. Automatic passage is performed according to the algorithm preset with a defined density, which is calculated using a monotone image created by the integration of serial images from over-focus to under-focus (Additional figure 3C and D).

Additional figure 4 shows the typical duration of each operation, such as the preparation of reagents and sample dish, medium change, digestion, seeding, and clean-up in case of a typical 10-cm culture plate (Additional Movie). Culture parameters, such as the duration of the digestion for cell harvest, tapping frequency, strength of the digestion, volume of the medium, and centrifugation conditions (gravity, temperature, and duration), are programmable.

Successive culture of human iPS (MRC5; RCB0211) cells for 15 passages (approximately 4 months) was conducted, which was reported in a poster presentation at the 9th Annual Meeting of International Society of Stem Cell Research in Boston in June 2013. After culture for 15 passages, the cells were confirmed to be undifferentiated and pluripotent, having normal karyotypes. Many cell lines, including CHO, HeLa, NIH3T3, HEK293, Caco-2, PC12, HepG2, COS-7, A375, and IMR-32, have been cultured using this method and compared with a manual culture method.

A user-friendly schedule management software is also part of the system. Additional figure 5 shows an image of the screen of the software. The system estimates the amount of consumables to be used, predicts the time they will become short, and highlights schedules with a color where consumables may be in short supply.

**Additional Figure Legends**

Additional figure 1: Software applications and relationship

| **Application** | **Main Features** | **Installed Hardware** |
| --- | --- | --- |
| Centralized Monitoring System/Process Management System | Conducts overall monitoring of the machine, including device conditions and operation progress. Manages operation process and operation results. In addition, provides MMI to users. | Control PC |
| Operation Management System | Sends movement directives to devices in accordance with the operation orders given by the Process Management System. | Control PC |
| Robot Control System | Controls robot, external shaft, and hand in accordance with the movement directives. | Robot controller |
| Image Processing System | Controls visual devices in accordance with the movement directives. Provides the Centralized Monitoring System with cell images. | Image processing PC |
| Device Control System | Controls devices in accordance with the movement directives. Conducts various local operations through a touch panel installed in the machine. | PLC |

MMI: Man–Machine Interface


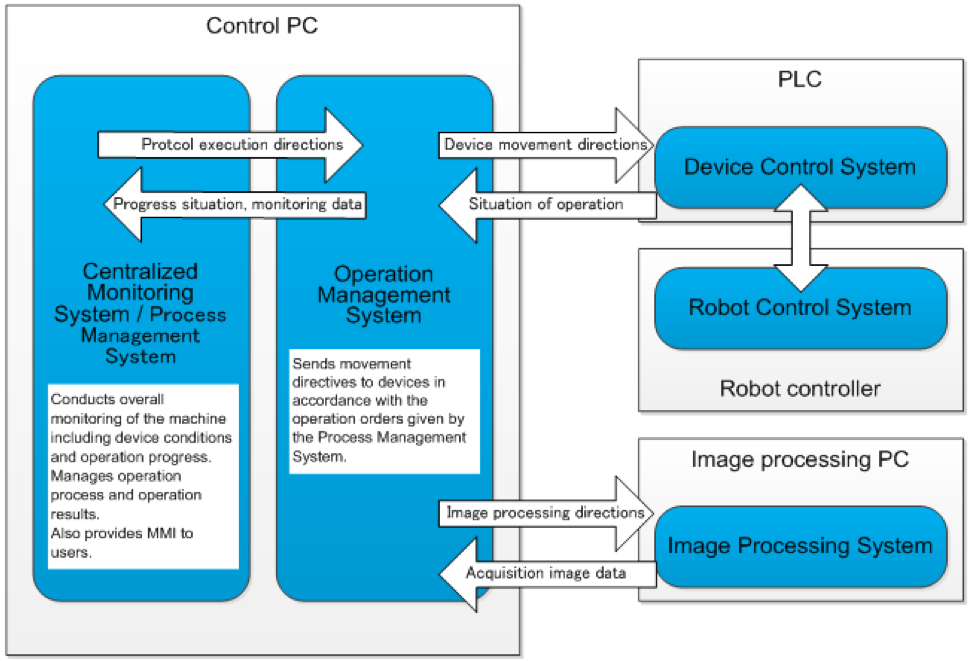


Additional figure 2: Machine reliability. (A) Disposable tip, (B) Sample label, (C) Alarm system, (D) Monitoring display


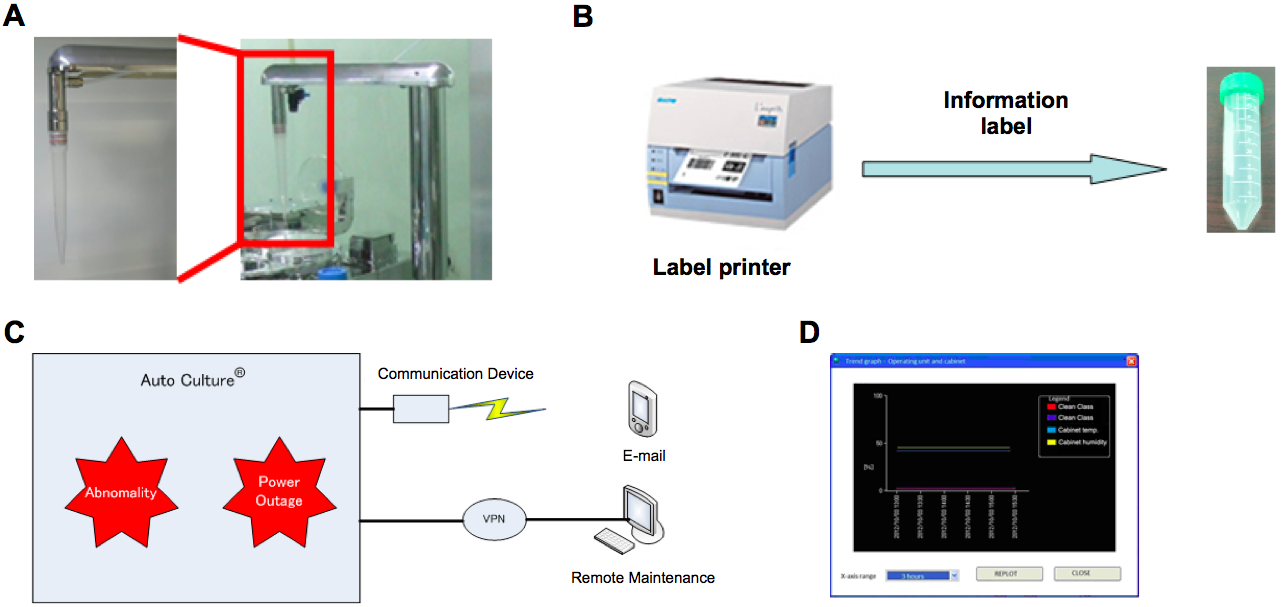


Additional figure 3: Morphological observation. (A) Representative serial photographs obtained in a previous experiment. (B) External monitor display, (C) Phase contrast microscope, (D) Integration into a monotone image to calculate the cellular density


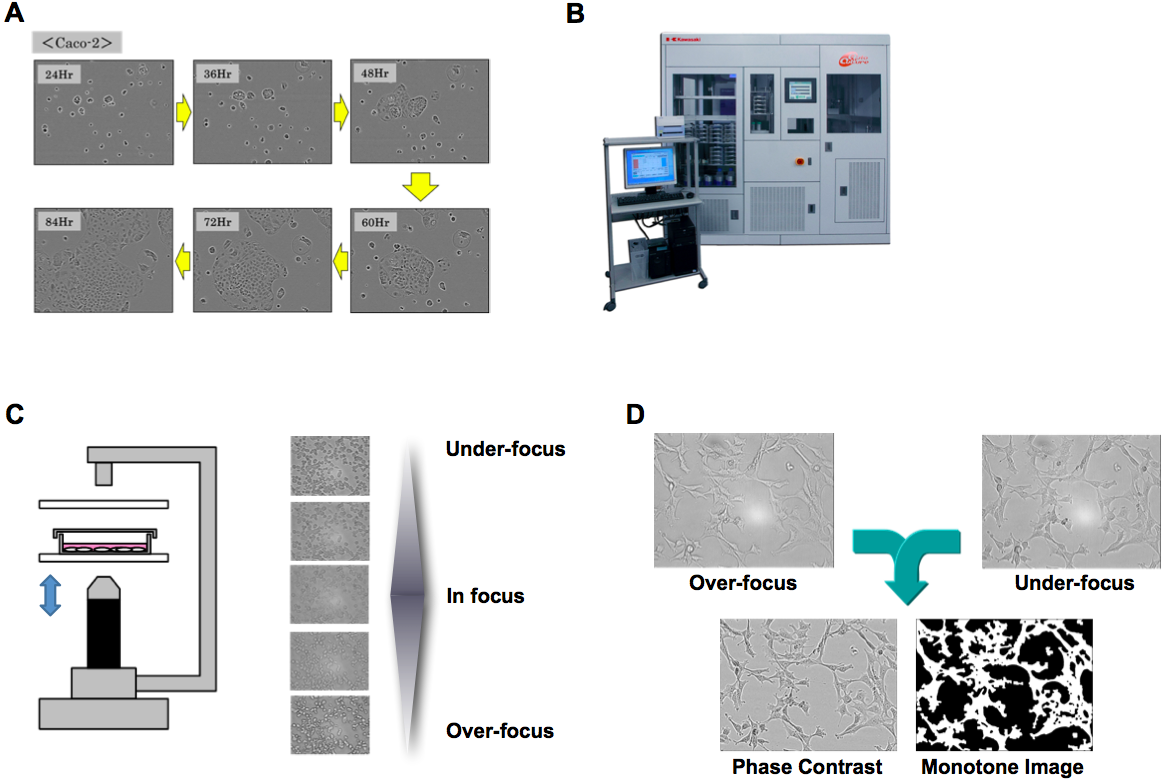


Additional figure 4: Typical duration of each cell culture operation

| **Operation Procedure** | **Approximate Time Required (min)** | |
| --- | --- | --- |
|  | **1 plate → 1 plate** | **1 plate → 5 plate** |
| **Preparation** | 3 | |
| **Medium removal** | 3.5 | |
| **PBS cleaning** |  |  |
| **Cell detachment** | 10 | |
| **Cell collection** |  |  |
| **Centrifugation** | 5 | 8 |
| **Supernatant disposal** | 5 | 15.5 |
| **Re-suspension** |  |  |
| **Seeding** |  |  |
| **Clean-up** | 4 | |
| **Total** | 30.5 | 44 |

Additional figure 5: Schedule management software display


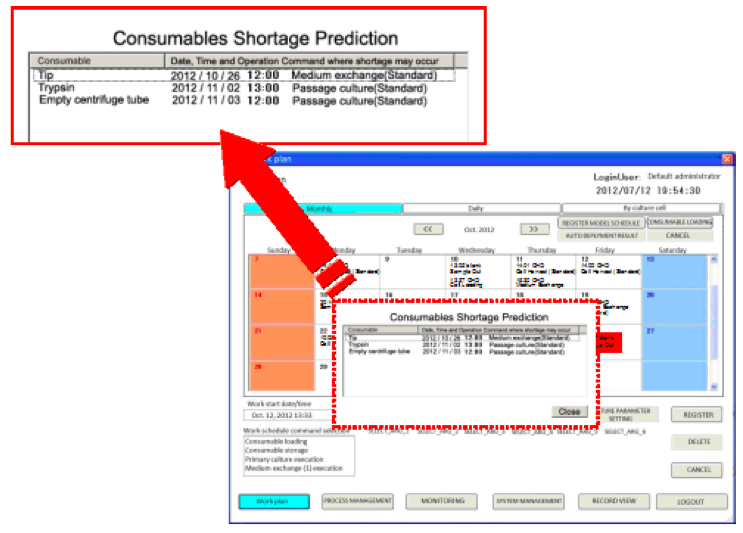

Supplement: Additional file 1 — Document 1. Specialization of the automated cell processing machine (Auto Culture®). [file 1472-6750-13-102-S1.docx]
