# Supplementary material for: Large-scale cell production of stem cells for clinical application using the automated cell processing machine
Source: BMC Biotechnol. 2013 Nov 15;13:102. doi: 10.1186/1472-6750-13-102 (PMC4101824; doi:10.1186/1472-6750-13-102)
Supplement: Additional file 2 — Document 2. Quantitative cellular aspects for ALCADIA clinical trial. [file 1472-6750-13-102-S2.docx]

Additional Document 2

**Quantitative cellular aspects of the ALCADIA clinical trial**

Six patients were enrolled in the ALCADIA clinical trial, and cardiac stem cells were acquired for autologous cell transplantation. The average cell number for grafting was determined to be 3.59 × 10^7^. On day 0, immediately after tissue digestion of the biopsy samples, the average cell number was 2.1 × 10^5^. Cell passages were performed on days 11, 19, 27, and 36, and the total yields of cells were 8.0 × 10^5^, 7.8 × 10^6^, 1.358 × 10^7^, and 4.117 × 10^7^, respectively. The seeded cell numbers in each passage were 1.7 × 10^5^, 1.5 × 10^5^, and 8 × 10^4^, respectively. Doubling times of each cell population following the first, second, and third passage were 1.45, 1.23, and 0.89 days, respectively. After the first passage, the cardiac stem cells were considered to have entered into the exponential phase. Based on these doubling times in the exponential phase, the predicted time to reach to the desired cell number (3.59 × 10^7^) for grafting was calculated using the following equation:

(Predicted Time) = (Doubling time of the population) × ln (X_exit_/X_entry_)/ln2

X_exit_; the desired cell number (3.59 × 10^7^ in this experiment), X_entry_; the cell number to be seeded following cell passage

For P1, P2, and P3 cell populations, the values were 12.77, 10.84, and 7.82 days, respectively. We set the duration of cell culture to 2 weeks to obtain a sufficient number of cells for implantation into the patient’s heart.
